# Partial male-to-female reprogramming of mouse fetal testis by Sertoli cell ablation

**DOI:** 10.1242/dev.201660

**Published:** 2023-07-17

**Authors:** Kenya Imaimatsu, Ryuji Hiramatsu, Ayako Tomita, Hirotsugu Itabashi, Yoshiakira Kanai

**Affiliations:** Department of Veterinary Anatomy, The University of Tokyo, Yayoi 1-1-1, Bunkyo-ku, Tokyo 113-8657, Japan

**Keywords:** FOXL2, FGF9, AMH, Toxin receptor-mediated cell knockout, *Ex vivo* system

## Abstract

Temporal transcription profiles of fetal testes with Sertoli cell ablation were examined in 4-day culture using a diphtheria toxin (DT)-dependent cell knockout system in *AMH-*TRECK transgenic (Tg) mice. RNA analysis revealed that ovarian-specific genes, including *Foxl2*, were ectopically expressed in DT-treated Tg testis explants initiated at embryonic days 12.5-13.5. FOXL2-positive cells were ectopically observed in two testicular regions: near the testicular surface epithelia and around its adjacent mesonephros. The surface FOXL2-positive cells, together with ectopic expression of *Lgr5* and *Gng13* (markers of ovarian cords), were derived from the testis epithelia/subepithelia, whereas another FOXL2-positive population was the 3βHSD-negative stroma near the mesonephros. In addition to high expression of *Fgfr1*/*Fgfr2* and heparan sulfate proteoglycan (a reservoir for FGF ligand) in these two sites, exogenous FGF9 additives repressed DT-dependent *Foxl2* upregulation in Tg testes. These findings imply retention of *Foxl2* inducibility in the surface epithelia and peri-mesonephric stroma of the testicular parenchyma, in which certain paracrine signals, including FGF9 derived from fetal Sertoli cells, repress feminization in these two sites of the early fetal testis.

## Teaser

**Summary:** The paracrine actions of fetal Sertoli cells are crucial to repress feminization in surface epithelia/subepithelia and peri-mesonephric interstitial tissues, even after testis formation in the mouse embryo.

## INTRODUCTION

In mouse early gonadogenesis, the coelomic epithelia covering the mesonephric region are crucial for gonadal formation as a source of gonadal somatic cell precursors that are recruited in a stage-dependent manner (DeFalco et al., 2011; Harikae et al., 2013a; Karl and Capel, 1998). By embryonic day (E) 11.5, the gonadal surface epithelial cells proliferate and form the primary sex cords, including supporting cell progenitors and germ cells in both male and female gonads. In the male gonads, the transient expression of sex-determining region Y (*Sry*) from E10.5 to E11.5 triggers the differentiation of these bipotential supporting cells into SOX9-positive Sertoli cells (see review by Kanai et al., 2005; Kashimada and Koopman, 2010). These differentiating SOX9-positive Sertoli cells produce several key autocrine/paracrine signals for the maintenance and organization of Sertoli cells into well-defined testis cords (Hiramatsu et al., 2010; Kim et al., 2006), for the mesonephric cell migration of vascular and perivascular progenitor cells (Coveney et al., 2008; Kumar and DeFalco, 2018; Martineau et al., 1997), and for Leydig cell differentiation in interstitial and peri-mesonephric stromal regions in the fetal testes at E12.5 (Brennan et al., 2003; Yao et al., 2002). Indeed, at E11.5, pre-Sertoli cells produce fibroblast growth factor 9 (FGF9), together with prostaglandin D2, to maintain their high SOX9 expression (Hiramatsu et al., 2009; Kim et al., 2006; Moniot et al., 2009; Wilhelm et al., 2007), which also induces male-specific epithelial proliferation and peri-mesonephric/vascular cell migration underneath the surface epithelia and inter-cordal spaces (Colvin et al., 2001; Combes et al., 2009; Schmahl et al., 2004). At E12.0-12.5, the differentiated Sertoli cells express platelet-derived growth factor (PDGF; Brennan et al., 2003) and desert hedgehog (DHH; Yao et al., 2002) to trigger differentiation of 3βHSD-positive Leydig cells from interstitial stroma cells (see review by Svingen and Koopman, 2013). Anti-Müllerian hormone (AMH) is involved in mesonephric/vascular cell migration (Ross et al., 2003), Müllerian duct regression (Behringer et al., 1990, 1994) and the maintenance of Sertoli cells in the testis cords in cooperation with activin (Rodriguez et al., 2022). Testis-specific mesonephric/vascular cell migration may disrupt the direct connection between surface epithelia and primary sex cords (see review by Harikae et al., 2013a; Suzuki et al., 2015), leading to tunica albuginea and interstitial regions being outside the testis cords at E12.5-13.5. Therefore, the paracrine actions of Sertoli cells are crucial for establishing the principal testis architecture at the fetal stage.

In ovarian primary sex cords, supporting cells do not express *Sry*/*Sox9* (Albrecht and Eicher, 2001), but do express *Foxl2*, an ovary-determining factor in goat, at E12.0-12.5 in mice (Boulanger et al., 2014; Schmidt et al., 2004). These FOXL2-positive cells are mitotically arrested pre-granulosa cells (Gustin et al., 2016; Mork et al., 2012) that contribute to the first wave of folliculogenesis soon after birth (see review by Imaimatsu et al., 2022; Suzuki et al., 2015). In fetal ovaries at E12.5 and thereafter, the surface epithelia continuously undergo proliferation, ingression and expansion into the subepithelial region (Mork et al., 2012; Rastetter et al., 2014), leading to the formation of FOXL2-positive ovarian cords (also known as ‘ovigerous’ or ‘secondary sex’ cords) throughout the late fetal stages (Ng et al., 2014; Niu and Spradling, 2020; Rastetter et al., 2014; Suzuki et al., 2015). The ovarian cords, including some surface epithelial cells, are marked by female-specific expression of the leucine-rich repeat-containing G-protein-coupled receptor 5 (*Lgr5*) and guanine nucleotide-binding protein (G protein), gamma 13 (*Gng13*), and contribute to the resting primordial follicles to sustain fertility throughout life (Fujino et al., 2007; Niu and Spradling, 2020; Rastetter et al., 2014; Zheng et al., 2014). Taken together, the above suggest distinct sex-dimorphic cellular events in the surface epithelia between the fetal testis and ovary at E12.5 and later stages. However, the mechanisms are unclear.

Toxin receptor-mediated cell knockout (TRECK) mice that carry a human diphtheria toxin (DT) receptor (HBEGF-mut) transgene driven by a cell linage-specific promoter can achieve conditional loss of lineage-specific cells by the administration of DT (Saito et al., 2001). *AMH-*TRECK transgenic (Tg) mice carrying the *AMH* promoter-driven DT receptor can be used for conditional ablation of AMH-positive gonadal supporting cells by DT treatment *in vivo* and *in vitro* (Shinomura et al., 2014; Rebourcet et al., 2014). Previous studies revealed the *in vivo* roles of the fetal and postnatal Sertoli cells at and after E14.5 by using the Sertoli cell ablation in combination with *AMH*-Cre and Cre recombinase-inducible DT fragment A (DTA) (Rebourcet et al., 2014; Wang et al., 2020). In this study, we focused on the paracrine effects of differentiated Sertoli cells on other testicular somatic cells at E12.5-14.5. *Ex vivo* direct treatment of fetal *AMH-*TRECK Tg testes with DT led to Sertoli cell depletion and ectopic FOXL2 expression in testicular somatic cells near surface epithelia and mesonephros, suggesting an unexpected male-to-female sex reversal model of E12.5 testes. Such ectopic FOXL2 induction in fetal testes may be limited to initiation at E12.5-13.5, and its potency was reduced by exogenous FGF9, implicating FGF9 loss in sex reversal in the surface epithelia/subepithelia and peri-mesonephric stroma in early fetal testes with Sertoli cell ablation.

## RESULTS

### DT-dependent Sertoli cell ablation in AMH-TRECK Tg testes *ex vivo*

**Fig. 1.**
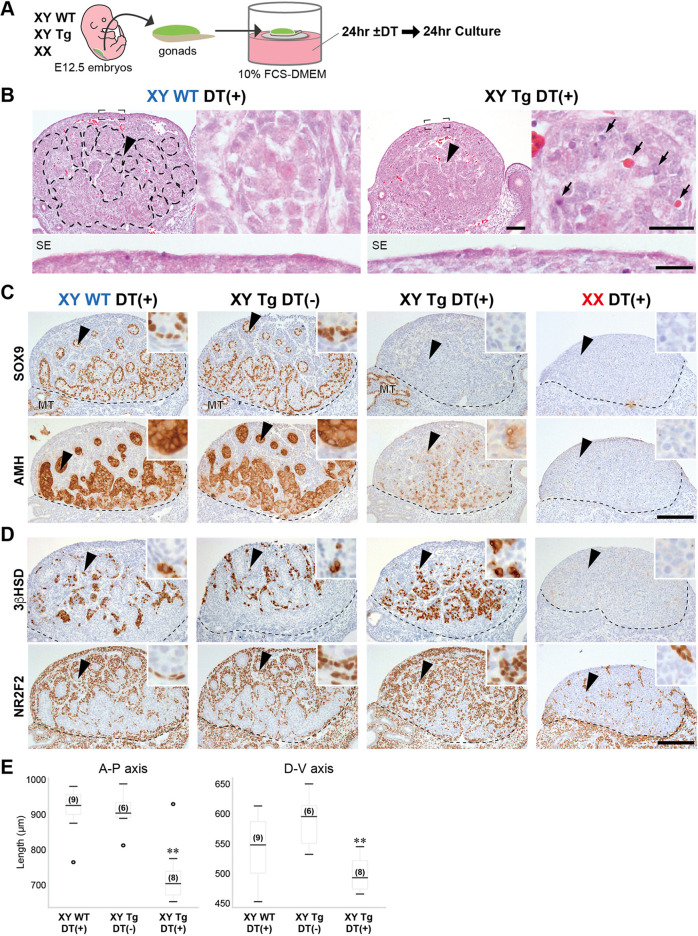
**Sertoli cell ablation of E12.5 testes in *AMH*-TRECK transgenic (Tg) mice.** (A) Testis/ovary explants isolated from Tg and wild-type littermates at E12.5 were treated with diphtheria toxin (DT; 200 ng/ml) for 24 h and then recovered for 24 h in FCS-DMEM. (B) Hematoxylin and Eosin staining of DT-treated wild-type and Tg testis explants, showing degenerated testis cords, including pyknotic nuclei and necrotic cell debris (arrows), but histologically normal surface epithelia/subepithelia (SE; lower panels), in the Tg explant. Dashed lines indicate testis cords. (C,D) Anti-SOX9 (a marker of Sertoli cells and mesonephric tubules), -AMH (a Sertoli cell marker), -3βHSD (a Leydig cell marker) and -NR2F2 (a marker for stromal cells) immunostaining (brown) of wild-type and Tg testes/ovaries with (+) or without (−) DT treatment, showing a marked reduction of SOX9- and AMH-positive signals in DT-treated Tg testes (C). 3βHSD- and NR2F2-positive signals were unaltered in DT-treated Tg testes (D). Broken lines indicate the border between gonad and mesonephros. (E) Morphometric analysis showed a significant reduction in testis size (A-P or D-V axes) in DT-treated Tg testes compared with the two control testis samples (**P<0.01; one-way ANOVA followed by Dunnett's test). Box and whisker plots show medians, interquartile ranges (boxes), and minima and maxima (whiskers). Numbers in parentheses are the numbers of samples. Arrowheads indicate the area magnified on the right in B, and in the insets in C and D. MT, mesonephric tubule. Scale bars: 100 µm in B (top left images); 25 µm in B (top right and lower images); 200 µm in C and D.

In developing mouse testes, differentiated Sertoli cells in the testis cords start to express *Amh* at E12.5 (Münsterberg and Lovell-Badge, 1991). In this study, to examine the paracrine function of fetal Sertoli cells at E12.5 and thereafter, we isolated the testes/ovaries of wild-type and Tg embryos and treated them with DT (200 ng/ml) in 24 h organ culture for Sertoli cell ablation (Fig. 1A-E). After 24 h recovery culture in FCS-DMEM, the phenotypes of gonadal explants were examined by morphometric and immunohistochemical analyses (Fig. 1B,E). Histological analysis revealed that, in wild-type testes, DT treatment induced no appreciable defect in the testis cords (Fig. 1B). By contrast, in the Tg testes, DT treatment caused severe deformation of the testis cords, in which nuclear debris and necrotic death of Sertoli and germ cells were found throughout the presumptive testis cord region. This is in contrast to the normal cell morphology in the surface epithelial and interstitial regions around the deformed testis cords (Fig. 1B). Moreover, morphometric analysis confirmed a significant reduction in the length of the anterior-posterior or dorsal-ventral axis of DT-treated Tg testes compared with DT-treated wild-type and non-treated Tg (control) testes (Fig. 1E).

Anti-SOX9 and AMH immunostaining showed that SOX9- and AMH-positive Sertoli cells formed the well-defined testis cords in the parenchyma of DT-treated wild-type and non-treated Tg control testes (Fig. 1C). In Tg testis explants, DT treatment caused complete loss of SOX9-positive signals in the testicular parenchyma, despite intact SOX9-positive signals in the mesonephric tubules (Fig. 1C). Moreover, most of the anti-AMH-positive signals were reduced in DT-treated Tg explants. In contrast to Sertoli cell depletion in Tg explants, the 3βHSD-positive Leydig cells and NR2F2-positive stromal cells were intact in the presumptive tunica albuginea, and in the interstitial and peri-mesonephric regions of DT-treated Tg testes (Fig. 1D). Therefore, the DT-treated Tg testis explants showed almost complete ablation of Sertoli cells without appreciable damage to the other gonadal soma; e.g. tunica albuginea and interstitium, at E12.5. This *ex vivo* system enables evaluation of the paracrine actions of fetal Sertoli cells at and after E12.5 *ex vivo*.

In addition, TUNEL staining revealed that some apoptotic cells were still detectable 1 day culture after DT treatment (Fig. S1A,B), but TUNEL-positive signals, as well as AMH-positive and SOX9-positive signals, were rarely observed at 4-day culture of DT-treated Tg explants (Fig. S1C,D), indicating the complete depletion and no reappearance of Sertoli cells in Tg testes after DT treatment.

### Temporal alterations of the RNA transcriptome in 4-day culture of DT-treated Tg testes

**Fig. 2.**
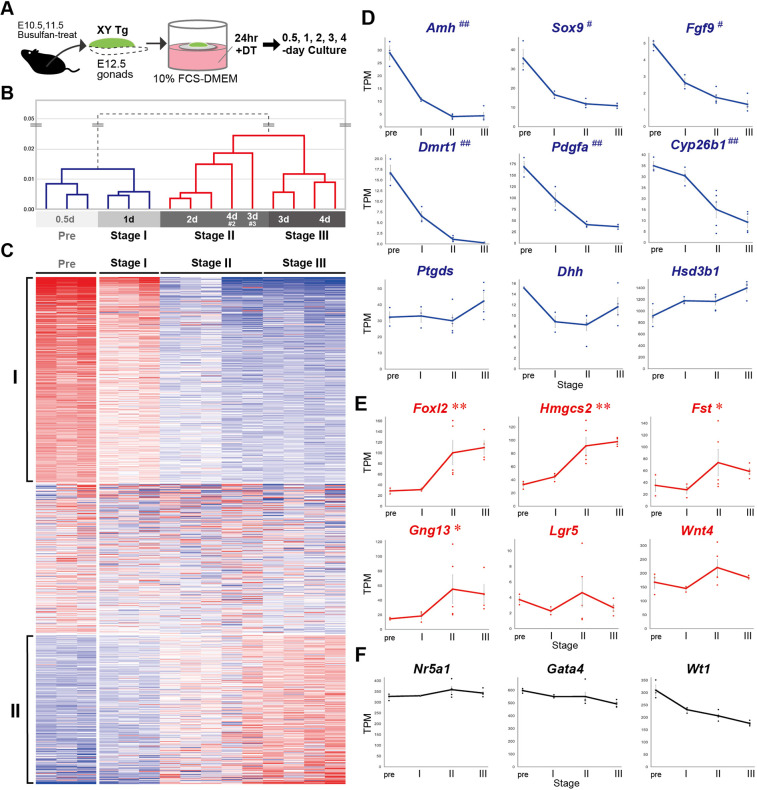
**Temporal transcriptome alterations of testis or ovary-specific genes in a 4-day culture of Tg testes after 24 h of DT treatment.** (A) Testes without the adjacent mesonephros were isolated from E12.5 Tg embryos pretreated with busulfan. They were treated with diphtheria toxin (DT; 200 ng/ml) for 24 h, cultured for 0.5, 1, 2, 3 and 4 days, and subjected to RNA-seq analysis. (B) Classification of four stages (Pre, I, II and III) by hierarchical clustering using transcripts per million (TPM, normalized RNA-seq read counts) of bulk RNA-seq data from Tg testis explants at 0.5-, 1-, 2-, 3- or 4-day culture (three sets each) after 24 h DT treatment. (C) Heatmap of K-mean clustering using the top 2000 genes with altered expression at each stage; 750 genes in cluster I and 546 in cluster II were down- and upregulated, respectively. Signal intensity: high, red; medium, white; low, blue. (D-F) Relative expression levels of marker genes (TPM from bulk RNA-seq data, mean±s.e.m. on the *y*-axis) at each stage (*x*-axis). # or ## in the gene symbol indicates significantly altered transcript levels in stage III compared with pre or both pre and stage I, respectively [log_2_(fold-change)<−1, modified *P*<0.05]. Asterisks in the gene symbol indicates significantly altered transcript levels in stage III (*) or both stage II and III (**), respectively [log_2_(fold-change)>1, compared with stage I; modified *P*<0.05].

Next, we monitored the transcription profile in 4-day culture of fetal testes with Sertoli cell ablation. DT-treated Tg testes at E12.5 were cultured for 0.5, 1, 2, 3 or 4 days, and three sets per culture period (each set had at least four testis explants) were subjected to bulk RNA-seq analysis (Fig. 2A).

Hierarchical clustering of 15 RNA-seq datasets revealed that the transcription profiles could be divided into 0.5/1-day and 2/3/4-day culture periods in DT-treated Tg testes (Fig. 2B), suggesting dynamic transcriptional alterations in Tg testes at 1 and 2 days after 24 h DT treatment. Moreover, based on the transcriptome similarity, all sample sets were classified into the following four stages: pre-stage (three sets of 0.5-day culture samples), stage I (three sets of 1-day samples), stage II (three sets of 2-day samples, one set each of 3-day and 4-day samples) and stage III (two sets of 3-day and 4-day samples). k-Mean gene clustering analysis showed the presence of two major clusters: 750 stage-dependently transcript-reduced genes (cluster I) and 546 stage-dependently transcript-increased genes (cluster II) (Fig. 2C, Table S1A,B). In cluster I, reduced expression of Sertoli cell-specific genes, such as *Amh*, *Sox9*, *Fgf9*, *Dmrt1*, *Pdgfa* and *Cyp26b1* (Bowles et al., 2006; Brennan et al., 2003; Colvin et al., 2001; da Silva et al., 1996; Kent et al., 1996; Münsterberg and Lovell-Badge, 1991; Raymond et al., 1999), was detectable with significant transcript reduction (Fig. 2D), suggesting Sertoli cell ablation in Tg explants. By contrast, several key genes crucial for the paracrine actions of Sertoli cells, such as *Dhh* (encoding hedgehog signals to induce Leydig cell differentiation; Bitgood et al., 1996; Yao et al., 2002) and *Ptgds* (encoding the PGD_2_ synthase; Adams and McLaren, 2002), as well as *Hsd3b1* (a Leydig cell marker; Baker et al., 1999), showed no appreciable alteration in expression in DT-treated testis explants (Fig. 2D); this is likely due to the rapid reduction of their transcripts in the damaged Sertoli cells, with their levels reaching a minimum by 1 day after 24 h DT treatment (Fig. S2).

Surprisingly, in cluster II, pre-granulosa cell-specific genes, such as *Foxl2*, *Hmgcs2* and *Fst* (Bagheri-Fam et al., 2020; Loffler et al., 2003; Menke and Page, 2002; Yao et al., 2004), and a key ovarian cord marker, *Gng13* (Fujino et al., 2007; Niu and Spradling, 2020), were significantly increased, although there was no significant difference in the expression of key ovarian genes (*Lgr5* and *Wnt4*) in the RNA-seq data [Fig. 2E; Table S1B; see also quantitative RT-PCR (RT-qPCR) data in Fig. S3D]*.* Moreover, expression of *Wt1*, a marker of both gonadal supporting cells and surface epithelia in both sexes (Armstrong et al., 1993; Liu et al., 2016), was reduced slightly, albeit non-significantly (Fig. 2F). Expression of the gonadal somatic cell markers *Nr5a1* and *Gata4* was maintained (Fig. 2F), suggesting retention of *Wt1*-positive gonadal somatic cells in 4-day culture of Tg testes with Sertoli cell depletion. Taken together, these findings suggest male-to-female reprogramming of remaining testicular somatic cells in Tg testes with Sertoli cell depletion.

Differentially expressed gene (DEG) analysis of explants of stage II and III (relative to stage I) showed increased expression of 111 and 464 genes and decreased expression of 289 and 709 decreased genes, respectively [|log_2_(fold change)|>1 and adjusted *P-*value <0.05; Fig. S3A,B]. We next compared these DEGs with the testis- or ovary-specific genes at E12.5-13.5 (i.e. 2984 male and 2950 female genes expressed in a sex-dimorphic manner *in vivo*; Zhao et al., 2018). As a result, 154 of the 709 decreased DEGs in Tg testes at stage III were found among testis-specific genes *in vivo* (21.7%; 154/709 genes; Table S2A). Compared with increased DEGs at stage II/III, 35.1% (39/111 genes) at stage II, and 22.0% (102/464 genes) at stage III were found among the 2950 ovary-specific genes, including *Foxl2*, *Hmgcs2*, *Fst*, *Irx3* and *Gng13* (Fig. S3A,B; Table S2B,C).

RT-qPCR analyses of DT-treated Tg testis explants before and after a 4-day culture confirmed reduced expression of *Amh* and *Sox9* (Fig. S3C). Moreover, RT-qPCR analyses revealed a significant increase of *Wnt4* and *Lgr5* expression, early key ovarian-specific genes *in vivo* (Fig. S3D), in contrast to high variations of these two transcripts among the samples in bulk RNA seq data (see Fig. 2E). Taken together, these findings imply that DT-treated Tg testes undergo partial feminization *ex vivo*, which may be initiated by loss of Sertoli cells from the testes at E12.5. As a side note, the RT-qPCR data showed superior performance compared with bulk RNA-seq data for *Wnt4* and *Lgr5*. This was attributed to the advantage of RT-qPCR, which used a small RNA amount of each Tg or control explant, while bulk RNA-seq necessitated a substantial amount of RNA sourced from multiple gonads obtained from different pregnant mothers at various experimental dates.

### Appearance of FOXL2-positive signals near the surface epithelia/subepithelia and in the testicular interstitial stroma near the mesonephros

**Fig. 3.**
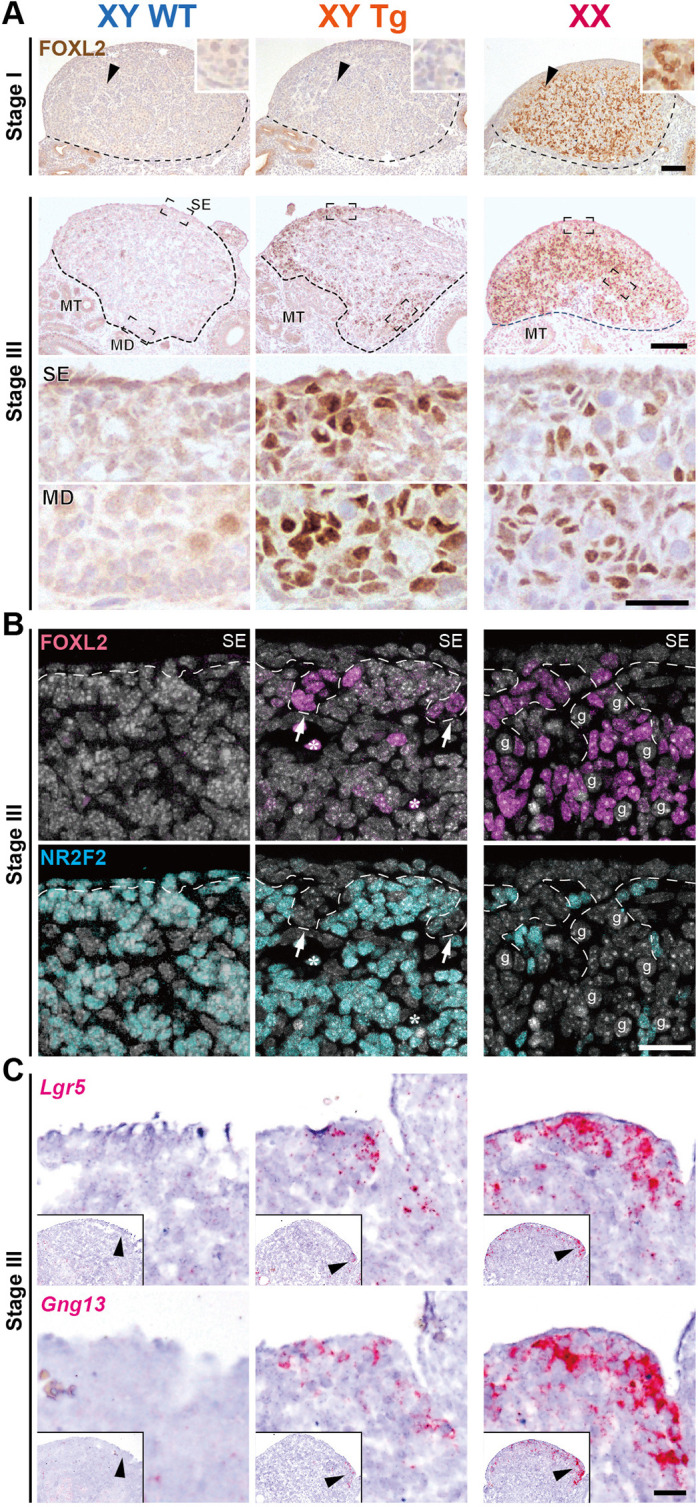
**Ectopic appearance of two distinct FOXL2-positive cells in the surface epithelia/subepithelia and peri-mesonephric regions in Tg testes with Sertoli cell ablation.** (A) Anti-FOXL2 immunostaining (brown) of DT-treated wild-type and Tg testes, and control ovaries before and after a 4-day culture following 24 h DT treatment, corresponding to stages I and III, respectively. Ectopic FOXL2-positive cells are seen both in the surface epithelia/subepithelia and the peri-mesonephric stroma. Broken lines indicate the border between gonad and mesonephros. Lower two panels at stage III are magnified images of the regions surrounded by broken rectangles in the upper panels. Arrowheads indicate the regions shown at higher magnification in the insets. (B) Anti-FOXL2 (magenta) and -NR2F2 (cyan) immunofluorescence (DAPI, white) of DT-treated wild-type and Tg testes, and ovaries showing FOXL2-positive cells (arrows) in surface cord-like structures (broken line) negative for NR2F2 (stromal cell marker) in DT-treated Tg testes. (C) *In situ* hybridization showing ectopic expression of *Lgr5* and *Gng13* in the surface epithelial/subepithelial region of DT-treated Tg testes and ovaries. Insets show the area shown at higher magnification (arrowheads) in the main image. Asterisks indicate autofluorescence of red blood cells. g, germ cells; MD, medullary peri-mesonephric region; MT, mesonephric tubules; SE, surface epithelium. Scale bars: 100 μm in A; 25 µm in B, C and lower two rows of A.

To evaluate the partial feminization of Tg testes after Sertoli cell ablation, we examined the spatial pattern of FOXl2 expression in DT-treated Tg testes before and after a 4-day culture (Fig. 3A). At 1 day after DT treatment, no FOXL2-positive signal was observed in Tg or wild-type testes (Fig. 3A; corresponding to stage I, as shown in the RNA-seq data; Fig. 2B). In a 4-day culture of Tg testes, corresponding to stage III, FOXL2-positive signals were found in somatic cells in the testicular parenchymal cells near the surface epithelia and mesonephros (Fig. 3A). Indeed, their FOXL2 signal intensity in Tg gonads was similar to that of the ovarian controls (insets in Fig. 3A). These findings suggest that Sertoli cell ablation of E12.5 testes resulted in the ectopic appearance of two distinct FOXL2-positive cell populations located near surface testis epithelia and in testicular stroma near the mesonephros.

In the surface subepithelial region of XY Tg explants in a 4-day culture, anti-FOXL2 immunostaining, together with anti-NR2F2 staining (a stromal cell marker that marks fetal gonadal somatic cells except Sertoli/granulosa and Leydig cells; McClelland et al., 2015; Rastetter et al., 2014), revealed FOXL2-positive subepithelial cells in the NR2F2-negative cell cluster that is directly associated with the surface epithelial layer (Fig. 3B). Such an NR2F2-negative/FOXL2-positive cord-like structure was also seen in the ovarian cords, including germ cells of female control explants, but not in the subepithelial region (which was replaced by the tunica albuginea), in the wild-type testis explants of a 4-day culture. Moreover, anti-FOXL2 immunostaining together with anti-WT1 antibody (a later marker for surface epithelium and supporting cells; Liu et al., 2016) revealed that some FOXL2-positive cells were located within the WT1-positive subepithelial cell clusters in the 4-day culture of DT-treated Tg testes (Fig. S4). These findings suggest the ectopic appearance of FOXL2-positive cells in the WT1-positive/NR2F2-negative ovarian cord-like structure in DT-treated Tg testes.

*In situ* hybridization revealed positive signals for *Lgr5* and *Gng13*, which are markers of ovarian cords (Niu and Spradling, 2020; Rastetter et al., 2014), in part of the epithelial and subepithelial regions of DT-treated Tg testes after a 4-day culture (Fig. 3C). These findings suggest that the FOXL2-positive ovarian cord-like structure may be ectopically induced in the surface epithelial region of testis explants after the removal of Sertoli cells.

**Fig. 4.**
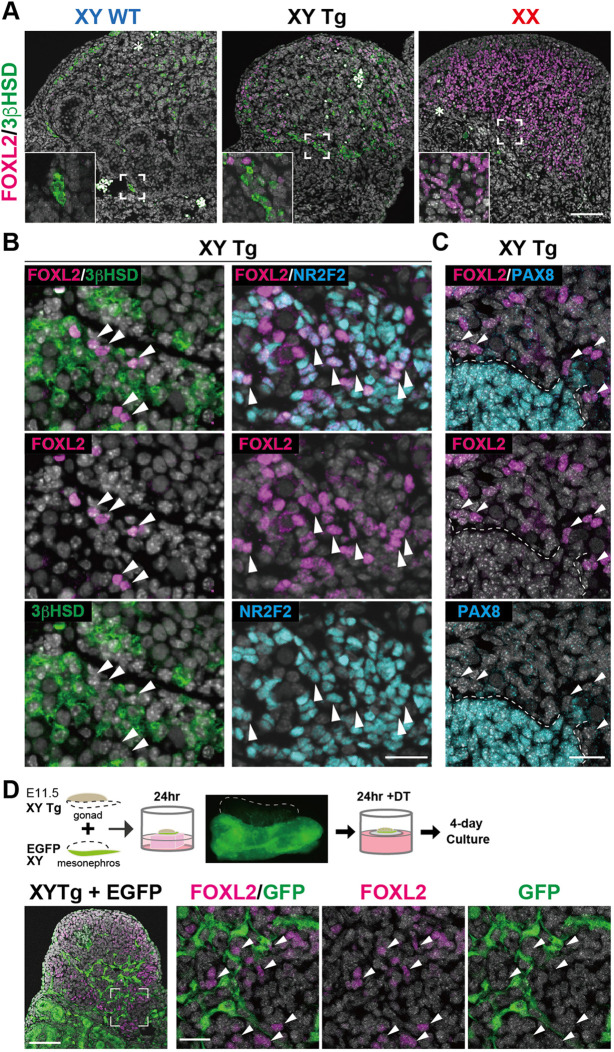
**Most FOXL2-positive cells are 3βHSD negative/NR2F2 positive in stroma of DT-treated Tg testes near the mesonephros.** (A,B) Anti-FOXL2 (magenta), -3βHSD (green) and -NR2F2 (cyan) immunofluorescence (DAPI, white) in DT-treated wild-type and Tg testes, and control ovaries in 4-day culture after DT treatment. 3βHSD-positive Leydig cells are distributed in the presumptive interstitial and peri-mesonephric regions of DT-treated Tg testes. Most FOXL2-positive cells (arrowheads) are 3βHSD-negative/NR2F2-positive stromal cells. (C) Anti-FOXL2 (magenta) and -PAX8 (cyan) immunofluorescence (DAPI, white) in DT-treated Tg testes in 4-day culture after DT treatment, showing no overlap of FOXL2-positive cells and PAX8-positive cells distributed at the border of the mesonephros. Broken lines indicate the border of the rete testis. (D) Schematic representation and a fluorescent dissecting microscopic image of the recombined explant culture of XY Tg gonad and CAG-EGFP-derived mesonephros at E11.5 (*n*=5). Anti-FOXL2 (magenta) and -GFP (green) immunofluorescence (DAPI, white), showing no overlap of FOXL2-positive cells and GFP-positive cells within DT-treated recombined Tg testis explants. Arrowheads indicate FOXL2-positive cells that are 3βHSD negative (left in B), NR2F2 positive (right in B), PAX8 negative (C) and GFP negative (D). Insets in A show high-magnification images of the regions surrounded by broken squares. Asterisks indicate autofluorescence of red blood cells. Scale bars: 100 μm in A and left-most panel in D; 25 µm in B, C and three right-most panels in D.

In the interstitial regions at the mesonephric side of Sertoli cell-ablated testes, FOXL2-positive signals were mainly found in NR2F2-positive cells and rarely in 3βHSD-positive Leydig cells (Fig. 4A,B). Moreover, anti-FOXL2 immunostaining, together with anti-PAX8 antibody (a marker for supporting-like cells and rete testis epithelia; Mayère et al., 2022; Uchida et al., 2022), confirmed no overlap in the expression of FOXL2 and PAX8 signals in the DT-treated testes (Fig. 4C). These data suggest that FOXL2-positive signals are present in certain interstitial stromal cells within the testicular parenchyma, rather than in the rete testis tissues located at the border of the mesonephros.

In XY gonads after E11.5, male-specific vascular/perivascular migration takes place from the adjacent mesonephros (Coveney et al., 2008; Martineau et al., 1997). These mesonephric cells then contribute to certain steroidogenic and smooth muscle cells, along with the formation of a vascular network, within the testicular interstitium (Kumar and DeFalco, 2018). Next, we used E11.5 XY Tg gonads combined with GFP-positive XY mesonephros at E11.5 (Hiramatsu et al., 2009). These XY gonads with GFP-positive mesonephros were cultured for 1 day to reach E12.5 testis development, including mesonephric migration. They were then treated with DT and cultured to recovery for 4 days to induce FOXL2-positive cells (upper scheme in Fig. 4D). In the DT-treated testes, GFP-positive cells originating from the mesonephros were observed in the testicular parenchyma. However, no detectable FOXL2-positive signals were found in the GFP-positive cells derived from the mesonephros in the DT-treated combined explants (Fig. 4D). These data suggest that the FOXL2-positive stromal cells in DT-treated Tg testes may originate from the gonadal parenchyma, rather than the mesonephros, before E11.5.

Finally, most germ cells degenerated inside the deformed testis cords (Fig. S5A), as in previous reports (Rebourcet et al., 2017; Shinomura et al., 2014). Interestingly, a subset of surviving germ cells initiated meiosis and was detected by staining for meiosis markers, including SCP3, REC8 and H2AFX (Fig. S5A,B). These findings align with the tendency towards increased transcript levels of these genes in the bulk RNA-seq analysis (Fig. S5C), further supporting the notion of meiotic initiation in these surviving germ cells.

### FOXL2-positive cells are recruited from proliferating epithelial and subepithelial cells in DT-treated Tg testes

**Fig. 5.**
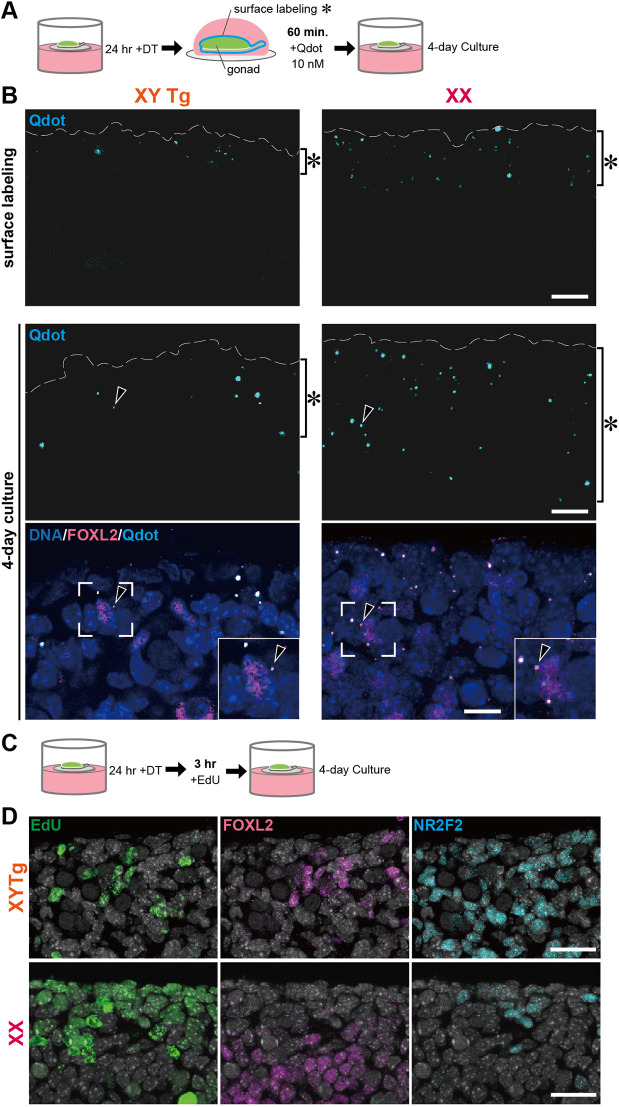
**Dynamics of surface FOXL2-positive cells in 4-day culture of Tg testis explants labeled with Qdot probe or EdU after 24 h of DT treatment.** (A) Schematic of the cell-labeling tracer experiment. E12.5 whole Tg testis or ovary attached to mesonephros was labeled with 10 nM Qdot probes for 1 h after 24 h of DT treatment, and cultured in FCS-DMEM for 4 days. (B) Immunofluorescence of the surface epithelia/subepithelia in DT-treated Tg testes before and after a 4-day culture. In Tg testis and ovary immediately after a 1 h incubation with Qdot probes, cytoplasmic Qdot signals (arrowheads) were restricted to only the surface epithelial region in the explants (bracket with asterisk). The apical epithelial surface is outlined by broken lines. Insets represent magnified images of the regions surrounded by broken squares. (C) Schematic of the EdU labeling experiment. E12.5 Tg explants were labeled with EdU for 3 h after 24 h of DT treatment and cultured for 4 days. (D) Anti-FOXL2 and NR2F2 immunofluorescence of DT-treated Tg testis pulse-labelled with EdU. Several FOXL2-positive/NR2F2-negative (non-stroma) cells were pulse-labeled with EdU in the surface region in the Tg testis explant (note the NR2F2-negative surface region of the control ovarian explant). Scale bars: 10 μm in B; 25 μm in D.

In fetal ovaries, the surface epithelia continuously proliferate throughout the fetal stages. The result is the ovarian cord structure, which subsequently forms the ovarian cortex, including primordial follicles (Gustin et al., 2016; Mork et al., 2012; Niu and Spradling, 2020). Next, to examine epithelial cell dynamics in DT-treated Tg testes, explant surfaces were labeled by Qdot probes (fluorescent nanocrystals) after DT treatment (Fig. 5A,B, asterisk), and the dynamics of labeled surface cells were traced in a 4-day culture. Cells with Qdot probes contribute to the thickening subepithelial region of DT-treated Tg testes, as in ovarian control explants (Fig. 5B). Anti-FOXL2 staining confirmed the presence of several FOXL2-positive cells with cytoplasmic Qdot probes in the subepithelial region, suggesting FOXL2-positive cells derived from the surface epithelia of the E12.5 testes. Moreover, a pulse labeling experiment using 5′-ethynyl-2′-deoxyuridine (EdU) for 3 h immediately after DT treatment revealed a cord-like cluster with EdU-positive cells in the epithelial/subepithelial region of DT-treated Tg testes, as in the ovarian control explants, albeit at higher density (Fig. 5C,D). In the surface epithelia/subepithelia of DT-treated Tg testes, these EdU-positive cell clusters were FOXL2 positive/NR2F2 negative, suggesting that the testicular epithelia proliferate and contribute to the surface FOXL2-positive cell population in Tg testes after Sertoli cell ablation.

### The ability to induce Foxl2 expression in the DT-treated Tg testes was lost by E14.5

**Fig. 6.**
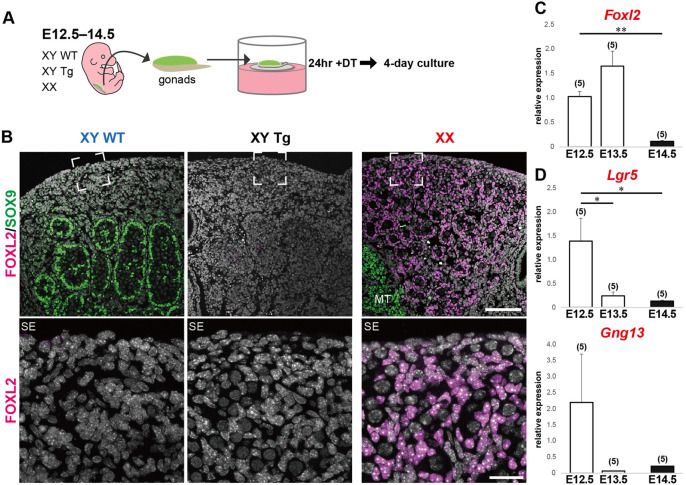
**Disappearance of DT-dependent FOXL2 inducibility in E14.5 Tg testes.** (A) Schematic of the experimental procedure. Testes isolated from Tg embryos and wild-type littermates at E12.5, E13.5 and E14.5 were cultured in FCS-DMEM for 4 days after a 24 h DT treatment. (B) Anti-FOXL2 (magenta) and -SOX9 (green) immunofluorescence showing no FOXL2-positive signals in the Tg testis explant with depletion of Sertoli cells (note no SOX9-positive signal in the DT-treated Tg testis). Lower panels are higher magnification images of the regions surrounded by broken squares in the upper panels. (C,D) RT-qPCR analysis showing significant loss of *Foxl2* expression (C), together with a lack of *Lgr5* and *Gng13* induction (D), in Tg explants initiated at E14.5 (*P<0.05, **P<0.01; one-way ANOVA followed by Dunnett's test). Numbers in parentheses are the numbers of samples. MT, mesonephric tubules; SE, surface epithelium. Scale bars: 100 μm (upper panels); 25 µm (lower panels).

Sertoli cell ablation of E12.5 testes caused ectopic *Foxl2* expression in the other testicular somatic cells, suggesting *Foxl2* inducibility is maintained in testicular somatic cells, except Sertoli cells, for some time after testis differentiation. To evaluate the time window, Tg testes were isolated at E12.5, E13.5 and E14.5, treated with DT for 24 h, and cultured for 4 days to undergo *ex vivo* gonadal development in the absence of Sertoli cells (Fig. 6A). After 4-day culture following DT treatment of Tg testes isolated at E14.5, SOX9-positive Sertoli cells were completely depleted in the testicular parenchyma, which also lacked FOXL2-positive signals (Fig. 6B). Moreover, RT-qPCR confirmed that the *Foxl2* inducibility in DT-treated Tg testes was maintained in those initiated at E13.5, but lost in those initiated at E14.5 (Fig. 6C). DT-dependent induction of the ovarian cord marker genes *Lgr5* and *Gng13* (Niu and Spradling, 2020) was reduced in these explants at E13.5 and E14.5, compared with E12.5. However, there was no significant difference in *Gng13* expression due to the marked variation among samples even at E12.5. Therefore, the *Foxl2* inducibility in the testicular soma is likely maintained at E12.5-13.5 but lost by E14.5.

### Exogenous FGF9 represses DT-dependent Foxl2 expression in E12.5 Tg testes *ex vivo*

**Fig. 7.**
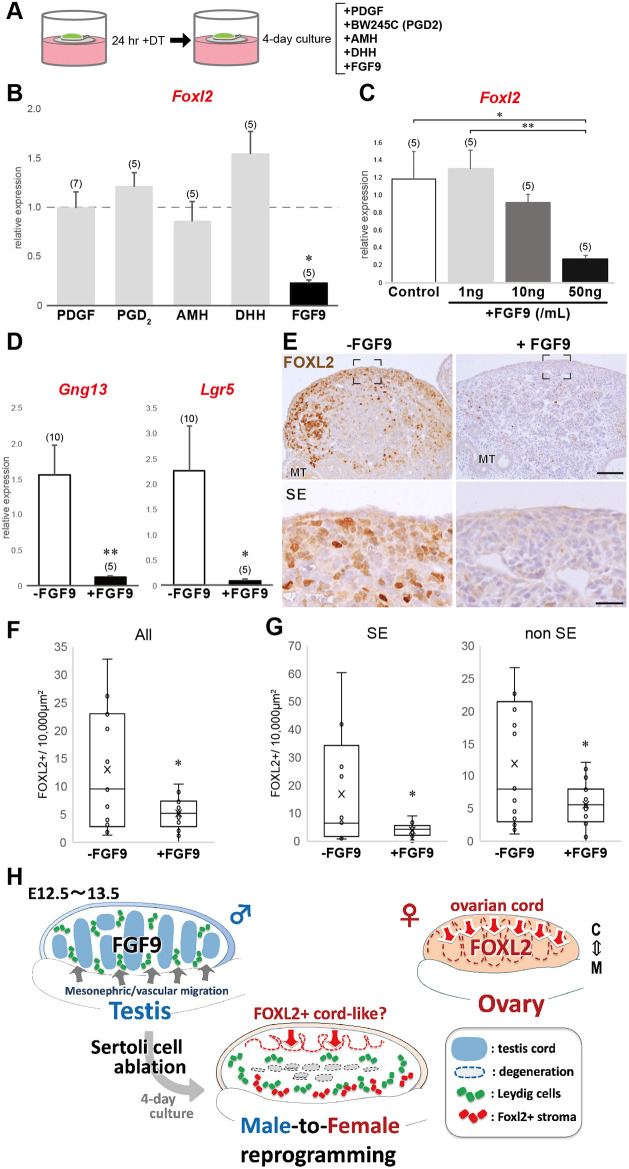
**Exogenous FGF9 is sufficient to repress FOXL2 inducibility in DT-treated Tg testis explants initiated at E12.5.** (A) Schematic of exogenous addition of a candidate ligand (PDGF, AMH, DHH or FGF9) or agonist (BW245C for PGD2) to a 4-day culture of DT-treated Tg testes. (B-D) RT-qPCR analysis showing significant reductions of *Foxl2* (B,C), and *Gng13* and *Lgr5* (D) in the presence of FGF9 (*P<0.05, **P<0.01; one-way ANOVA for FGF9, AMH and BW245C in B and C; paired Student's *t*-tests for others in B; unpaired Student's *t*-tests in D). Data are mean±s.e.m. In B, expression levels were normalized to the corresponding non-additive control of DT-treated Tg testes (broken horizontal line; set as 1.0). C shows relative *Foxl2* expression levels in DT-treated Tg testes cultured with FGF9 (0, 1, 10 or 50 ng/ml). *Actb* was used as an endogenous reference. Numbers in parentheses are numbers of samples. (E) Anti-FOXL2 immunostaining of DT-treated Tg testis explants in the presence or absence of FGF9. FOXL2-positive signals near the surface epithelia and mesonephros are repressed in Tg testes by exogenous FGF9. Lower panels are high-magnification images of the regions surrounded by broken rectangles in the upper panels. (F,G) Morphometric analysis of FOXL2-positive cell density [number per 10,000 µm^2^ in serial sections (each 40 µm apart) of a whole explant; *n*=3 per group] in DT-treated Tg testes cultured with or without FGF9. FOXL2-positive cells in the surface subepithelial region (SE) and the non-surface regions, including the peri-mesonephric site (non-SE), are enumerated in G (total FOXL2-positive cell density is shown in F). Data are presented as a box and whisker plot (median, center line; whiskers, sample distribution range; *P<0.05 by unpaired Student's *t*-test). Each point represents the data of one section (Mayère et al., 2022). (H) Schematic of the partial sex reversal of E12.5 testis after Sertoli cell ablation. In E12.5 testis, *Fgf9* expression in Sertoli cells, together with mesonephric/vascular cell migration, lead to testis differentiation (Harikae et al., 2013b). DT-dependent Sertoli cell ablation of E12.5 Tg testis triggers degeneration of the testis cord structure. In a 4-day culture of these testes without Sertoli cells, FOXL2-positive/NR2F2-negative cell clusters are re-recruited from the surface testis epithelia/subepithelia, similar to the ovarian cords from the female surface epithelia. FOXL2-positive/NR2F2-positive stromal cells appear in the peri-mesonephric region, independently of 3βHSD-positive Leydig cells in the interstitial region. Exogenous FGF9 represses FOXL2 expression in DT-treated Tg testes, implicating FGF9 in male differentiation of the surface epithelia/subepithelia and peri-mesonephric stromal cells. From top to bottom: cortex (C) to medulla (M) sides. MT, mesonephric tubule; SE, surface epithelium. Scale bars: 100 μm in E, upper panels; 25 µm in E, lower panels.

Sertoli cell ablation caused partial feminization in E12.5 testes, implicating the paracrine function of Sertoli cells in the maintenance of testicular somatic cells, especially the testicular surface epithelia. To identify the factors derived from Sertoli cells that modulate *Foxl2* inducibility, DT-treated Tg testes were cultured with PDGF (a promoter of mesonephric cell migration and epithelial cell proliferation; Brennan et al., 2003), BW245C (an agonist for the PGD2 receptor Dp1 with PGD2-like activity; an autocrine factor for Sertoli cell establishment; Malki et al., 2005; Moniot et al., 2009), AMH (a paracrine factor that promotes testis-specific mesonephric migration; Ross et al., 2003), DHH (a paracrine factor that promotes Leydig cell differentiation; Yao et al., 2002) and FGF9 (a key factor for Sertoli cell establishment, mesonephric migration and coelomic epithelial proliferation; Colvin et al., 2001; Kim et al., 2006; Schmahl et al., 2004) (Fig. 7A), all of which are involved in the male-specific pathway for testis formation during the sex differentiation period. Among these additives, only exogenous FGF9 repressed the DT-dependent induction of *Foxl2* expression in Tg explants (Fig. 7B). In addition to its dose-dependent repression of *Foxl2* inducibility in E12.5 testes (Fig. 7C), FGF9 also repressed expression of *Lgr5* and *Gng13* in DT-treated Tg explants (Fig. 7D). Anti-FOXL2 immunostaining and morphometric analysis of serial sections of testis explants confirmed a marked reduction of FOXL2-positive signals throughout the testicular parenchyma in DT-treated Tg testes cultured with FGF9 (Fig. 7E,F). Moreover, there was a significant reduction in the number of FOXL2-positive cells in the surface epithelial/subepithelial region or the remaining parenchyma, including the peri-mesonephric region (reduced levels: 23.2% in SE and 47.6% in non SE; Fig. 7G).

*In situ* hybridization confirmed that *Fgfr1* and *Fgfr2* (Kim et al., 2007; Schmahl et al., 2004), as well as anti-heparan sulfate proteoglycan (HSPG) (low-affinity receptors/reservoirs for FGF ligands; Harikae et al., 2013b), were highly expressed in the surface epithelial/subepithelial region and the peri-mesonephric region, in contrast to high *Fgf9* expression in Sertoli cells (Fig. S6A). Taken together with the RNA-seq data showing no altered *Fgfr1* and *Fgfr2* expression in 4-day cultures of DT-treated testes (Fig. S6B), the reduction in the FGF9 signal caused by Sertoli cell ablation may trigger the ectopic appearance of FOXL2-positive cells among remaining testicular somatic cells.

## DISCUSSION

Using the *AMH-TRECK* Tg line, we found that E12.5 testes with Sertoli cell ablation undergo ectopic induction of several ovary-specific genes, including *Foxl2*, *Irx3* and *Fst* (Jorgensen and Gao, 2005; Loffler et al., 2003; Menke and Page, 2002; Yao et al., 2004). This suggests that partial male-to-female reprogramming is induced in testicular somatic cells by the loss of Sertoli cells *ex vivo*. Two studies examined the testicular phenotypes of *in vivo* Sertoli cell ablation using *AMH*-Cre and Cre recombinase-inducible DT fragment A (DTA) or DT receptor (Rebourcet et al., 2014; Wang et al., 2020). However, Sertoli cell ablation in these testes *in vivo* could not be induced before E14.5, possibly owing to a considerable time lag in the death of Sertoli cells between the *in vivo* and *ex vivo* conditions. No male-to-female sex reversal phenotype, albeit of aberrant testicular vasculature and peritubular stroma, was observed in these fetal testes initiated after E14.5 *in vivo*. Together with the lack of DT-dependent FOXL2 inducibility in Tg testis initiated at E14.5 (Fig. 6), these data indicate that the cut-off time for FOXL2 inducibility is E13.5. This timing coincides with the establishment of the tunica albuginea, which is a thickened basement membrane layer underlying the surface epithelia and vasculature, after E13.5, as reported by Karl and Capel (1998).

In mouse testiculogenesis, all fetal Sertoli cells are recruited from the gonadal surface epithelia around E11.0-11.5 and tightly packed inside the well-defined testis cords, which are separated from the surface epithelia by E12.5 (Karl and Capel, 1998; see review by Harikae et al., 2013a). In DT-treated Tg testes at E12.5, FOXL2 was upregulated mostly in testicular somatic cell populations, except for the Sertoli cells, suggesting peripheral, not central, localization of ectopic FOXL2-positive cells at the surface epithelium and mesonephric border (Fig. 3A). This is in contrast to the sex reversal phenotypes of bipotential supporting cells in the primary sex cords/testis cords discovered using mutants of several key genes – *Rspo1*, *Wnt4* and *Ctnnb1* (Maatouk et al., 2008; see review by Chassot et al., 2014), *Dmrt1* (Matson et al., 2011; Zhao et al., 2015), *Znrf3* (Harris et al., 2018), *Nedd4* (Windley et al., 2022), and *Amh* and *Inhbb* (Rodriguez et al., 2022). Therefore, our DT-dependent FOXL2 inducible system using E12.5 Tg testes enables evaluation of feminization in testicular somatic cell components, except for the Sertoli cell lineage in early fetal testes.

In the female-type supporting cell populations, two major FOXL2-positive granulosa cell populations contribute to folliculogenesis in a cortical and medullary axis-dependent manner (Suzuki et al., 2015). One medullary population is bipotential supporting cells in the primary sex cords, which share an origin with fetal Sertoli cells in the testis cords (Albrecht and Eicher, 2001; Mork et al., 2012). The cortical population is the ovarian cords (i.e. secondary sex cords, which are continuously recruited from the ovarian epithelia at the late fetal and perinatal stages; Mork et al., 2012; Rastetter et al., 2014). These two distinct granulosa cell lineages were confirmed by a single-cell RNA sequence and lineage-tracing study (Niu and Spradling, 2020), in which *Gng13* and *Lgr5* expression marks the ovarian cords at the fetal stage in a female-specific manner (Niu and Spradling, 2020). In our *ex vivo* model of the fetal testis with Sertoli cell depletion, the FOXL2-positive/WT1-positive/NR2F2-negative cord-like structures were ectopically induced in the surface subepithelial region of DT-treated Tg testes (Fig. 3B, Fig. S4), together with *Gng13* and *Lgr5* upregulation in the testicular surface region (Figs 2E, 3C and Fig. S3D). Moreover, FOXL2-positive cells were, at least in part, derived from the proliferation of surface epithelial cells (Fig. 5). Therefore, such FOXL2-positive cells in the testicular surface region reflect the ectopic induction of ovarian cord-like FOXL2-positive cell clusters that are typically found in the ovarian cortical region at late fetal and perinatal stages (Mork et al., 2012; Niu and Spradling, 2020; Rastetter et al., 2014). This, in turn, suggests that fetal Sertoli cells repress ovarian cord formation in the surface epithelia, possibly via their paracrine actions, at E12.5-E13.5.

In DT-treated Tg testis explants, *Foxl2* inducibility, as well as *Lgr5* and *Gng13* expression, were repressed by exogenous FGF9 (Fig. 7B,D). FGF9 is a Sertoli cell-derived signal that induces mesonephric/vascular cell migration and coelomic vessel formation during early testis morphogenesis (Colvin et al., 2001), and maintains high SOX9 expression for Sertoli cell establishment in an autocrine manner (Hiramatsu et al., 2010; Kim et al., 2006). Genetic studies of FGF9-FGFR signals revealed male-to-female sex reversal in early fetal testes (Bagheri-Fam et al., 2017; Colvin et al., 2001; Jameson et al., 2012; Kim et al., 2006; Schmahl et al., 2004; Siggers et al., 2014). In fact, some *Fgf9*^−/−^ testes, albeit with various feminization phenotypes, showed reduced or depletion of subepithelial mesenchyme (Colvin et al., 2001), similar to the phenotype of ovarian cord-like formation in DT-treated Tg testes. *Fgfr1/2* and its mandatory co-factor HSPG were expressed in these two sites (Fig. S6A; Harikae et al., 2013b; Kim et al., 2007; Schmahl et al., 2004), thus Sertoli cell-derived FGF9 signals may contribute to repression of feminization in the surface epithelia and peri-mesonephric stroma in early fetal testes. This is consistent with the concept that FGF9 acts as an antagonist for WNT4-RSPO1 signals to regulate supporting cell establishment (Hiramatsu et al., 2009, 2010; Kim et al., 2006) via FOXL2 upregulation by RSPO1/WNT4/β-catenin signaling (Maatouk et al., 2008). Furthermore, the initiation of meiosis in certain surviving germ cells within DT-treated Tg explants (Fig. S5) is consistent with this finding. The absence of FGF9, which acts as an antagonist to retinoic acid, a meiosis initiation factor (Bowles et al., 2006, 2010; Koubova et al., 2006), further supports the role of FGF9 in regulating these processes.

In addition to FOXL2-positive supporting cell types, theca cells were also derived from at least two distinct FOXL2-positive progenitor populations: WT1-positive and -negative progenitors (Liu et al., 2015; Zhou et al., 2022). Although 3βHSD-positive theca cells were not detected in growing follicles by postnatal day (P) 14 *in vivo* (Zhou et al., 2022), some theca cells shared an origin with fetal Leydig cells in a *Nr5a1* promoter-EGFP line (Miyabayashi et al., 2015). The present study revealed that, in DT-treated Tg testes, most FOXL2-positive stromal cells in the peri-mesonephric region were 3βHSD-negative/NR2F2-positive/PAX8-negative cells that originated from the gonadal parenchyma, rather than the mesonephros, before E11.5 (Fig. 4). Based on the localization of ectopic FOXL2-positive cells with 3βHSD-positive Leydig cells (arrowheads in Fig. 4B), these FOXL2-positive stromal cells are similar to FOXL2-positive theca cell progenitors in fetal ovaries (Liu et al., 2015; Zhou et al., 2022). However, as the perivascular multipotent progenitors in the fetal ovary have been shown to contribute to granulosa, thecal and pericyte cell lineages (Li et al., 2022), the origin and fate of these FOXL2-positive stromal cells in DT-treated Tg testes warrant further research.

In conclusion, Sertoli cell ablation from early fetal testes induced the emergence of FOLX2-positive granulosa/theca-like progenitor cells in the surface epithelial and peri-mesonephric regions (Fig. 7H). Moreover, ectopic FGF9 addition repressed their FOXL2 expression. Our newly designed *ex vivo* system enables evaluation of the direct actions of signaling factors in testicular somatic cell components after Sertoli cell establishment in the early fetal testis.

## MATERIALS AND METHODS

### Animals

Animal experiments were carried out in accordance with the Guidelines for Animal Use and Experimentation of the University of Tokyo. The procedures were approved by the Institutional Animal Care and Use Committee of the Graduate School of Agricultural and Life Sciences of the University of Tokyo (approval ID: P18-046). The C57BL/6-Tg *AMH-*TRECK (toxin receptor-mediated cell knockout) male mice carrying human *AMH*-promoter-driven DT receptor-EGFP transgene (#94 line established by Shinomura et al., 2014) were mated with wild-type females (C57BL/6 or ICR strain; SLC) overnight and the next morning checked for the presence of a vaginal plug. Noon on the day when a vaginal plug was detected was considered embryonic day 0.5 (E0.5). Genotypic wild-type littermates were used as controls. C57BL/6-Tg (CAG-EGFP) mice (EGFP mice; SLC) were used for visualization of migrated mesonephric cells in combined culture with XY Tg gonads.

For RNA-sequencing (RNA-seq) analysis, pregnant females were pretreated with busulfan (intraperitoneal injection, 40 mg/kg, twice at E10.5 and 11.5) to eliminate the influence of germ cells and their transcripts on expression profiles in gonadal samples (Miura et al., 2019). We confirmed that the fetal testes of busulfan-treated embryos at E12.5 could induce ectopic appearance of FOXL2-positive cells in a similar manner to those in non-treated embryos in this Tg line by immunofluorescence (see Fig. S7).

### DT treatment and organ culture

At E12.5, fetal testes and ovaries were isolated from wild-type and Tg embryos in ice-cold phosphate-buffered saline (PBS). The sex and genotype of the isolated gonads were determined by their morphology and GFP fluorescence, in addition to genotyping PCR (Shinomura et al., 2014). Gonads were treated with or without DT (D0564-1MG; Sigma-Aldrich; 100-200 ng/ml) in 10% fetal calf serum (FCS)-Dulbecco's modified Eagle medium (DMEM; D5796; Sigma-Aldrich) for 24 h on ISOPORE membrane filters (TSTP02500; Millipore) at 37°C with 5% CO_2_. Next, the culture medium was exchanged for 10% FCS-DMEM, and the incubation was continued for up to 4 days, as described previously (Hiramatsu et al., 2003). To evaluate paracrine signals, Knockout Serum Replacement (KSR; 10828010; Gibco)-DMEM was used instead of FCS. DT-treated gonads were cultured in 10% KSR-DMEM supplemented with FGF9 (recombinant human fibroblast growth factor 9, F1168; Sigma; 50 ng/ml; Hiramatsu et al., 2009), BW245C [a hydantoin compound with prostaglandin D2 (PGD2)-like activity, 12050; Cayman Chemical; 50 nM], MIS/AMH (recombinant human MIS/AMH protein, 1737-MS-010/CF; R&D Systems; 100 ng/ml; Yamamoto et al., 2018), DHH (recombinant human desert hedgehog protein N-terminus, 4777-DH; R&D Systems; 100 ng/ml; Pathi et al., 2001) or PDGF-AA (recombinant human platelet-derived growth factor-AA, 165-25541; Fujifilm Wako; 100 ng/ml; Fujikawa et al., 2017) for 4 days. The medium was exchanged every 24 h.

### Bulk RNA-seq analysis

For RNA analysis, germ cell-depleted testes from busulfan-pretreated embryos were used as described previously (Miura et al., 2019). At E12.5, busulfan-pretreated testes were carefully separated from the adjacent mesonephros under a dissecting microscope. The testes were pretreated with DT in FCS-DMEM for 24 h, and incubated for 0.5, 1, 2, 3 and 4 days. Total RNA was extracted using TRIzol reagent (15596026; Invitrogen). Three sets of total RNA samples for each culture period (each set comprising four testes) were used to prepare RNA-seq libraries. The libraries were 50 bp single-end sequenced on an Illumina Novaseq6000 (Illumina). After removal of ribosomal RNA by mapping to the ribosomal RNA sequences using bowtie2 v. 2.3.4.1 (Langmead and Salzberg, 2012), sequences were mapped to the mouse genome (GRCm38/mm10) using STAR v. 2.6.0a (Dobin et al., 2013) and only uniquely mapped tags were extracted. Transcripts mapped to exons in each gene were quantified using featureCounts v. 1.6.2 (Liao et al., 2014).

Differential gene expression was analyzed using DESeq2 v. 1.18.1 (Love et al., 2014) in R (v. 3.4.3; R Development Core Team). Gene expression changes with |log2 fold change|>1.0 and adjusted *P*-value (Benjamini-Hochberg method) <0.05 were considered significant. The 2984 testis-specific and 2950 ovary-specific gene lists at E12.5-E13.5 (bulk RNA-seq data; Zhao et al., 2018) were compared with these differentially expressed genes. TPM counts were used as normalized counts for hierarchical clustering and temporal gene expression plots. Hierarchical clustering was conducted using the Python Scipy module. K-means clustering was conducted in the iDEP web application (Ge et al., 2018). The top 2000 genes ranked by standard deviations were divided into four clusters.

### Histology and protein immunodetection

Samples were fixed in 4% paraformaldehyde (PFA)/PBS and embedded in paraffin wax or OCT compound. Deparaffinized sections (4 μm) or cryosections (10 μm) were subjected to conventional histological (Hematoxylin-Eosin) or protein immunodetection. For immunohistochemical staining and immunofluorescence, sections were incubated with the primary antibodies listed in Table S3 in Tris-buffered blocking solution (TNB) for 12 h at 4°C. The reactions were visualized by incubation with biotin-conjugated secondary antibodies in combination with the Elite ABC Kit (PK-6100; Vector Laboratories) or Alexa-488/594/647 conjugated secondary antibodies listed in Table S3 with 4′,6-diamidino-2-phenylindore (DAPI). Stained sections were analyzed using an Olympus fluorescence microscope (BX51N-34-FL-2) and a Leica TCS SP8 confocal laser microscope.

### Morphometric analysis

To measure testis size, cultured explants were photographed using a dissection microscope (SZX16; Olympus) and the lengths of the long (anteroposterior) and short (dorsoventral) axes were measured using ImageJ Fiji (Schindelin et al., 2012). To quantify the number of FOXL2-positive cells, serial sections covering the entire testicular parenchyma were processed for immunostaining and photographed (×10 magnification). The number of FOXL2-positive cells per 10,000 µm^2^ was estimated separately in the surface epithelial and subepithelial (SE) regions, and in the other parenchymal (non-SE) region at the mesonephric side using 4 µm sections 40 µm apart (i.e. every 10 sections) in combination with the cell counter plug-in from Fiji (*n*=3 testes each).

### EdU labeling assay

To detect proliferating cells, gonadal explants were cultured in the presence of 20 µM 5′-ethynil-2′-deoxyuridine (EdU; 052-08843; Fujifilm Wako) for 3 h following 24 h DT treatment. The culture was continued in FCS-DMEM without EdU for 4 days. EdU labeling was visualized with the Click-iT Plus EdU Alexa Fluor 488 Imaging Kit (C10637; Invitrogen) according to the manufacturer's instructions. Paraffin wax sections of EdU-stained samples were co-stained using anti-FOXL2 and anti-NR2F2 antibodies, as described above.

### *In situ* hybridization

Samples were fixed with 10% neutral-buffered formalin and embedded in paraffin wax. Deparaffinized sections were processed for RNA *in situ* detection using the RNAscope Target Probe Mm-*Lgr5* (312171, NM_010195.2), *Gng13* (462531, NM_022422.5), *Fgf9* (499811, NM_013518.4), *Fgfr1* (443491, NM_010206.3) and *Fgfr2* (443501, NM_010207.2) with the RNAscope 2.5 HD Reagent Kit-RED System (ACDBio) as described previously (Imura-Kishi et al., 2021; Uchida et al., 2022).

### Quantitative RT-PCR

For quantitative RT-PCR (RT-qPCR) analysis, total RNA was purified from gonadal samples using TRIzol reagent (15596026; Invitrogen) and reverse-transcribed with Superscript VILO MasterMix (1175050; Invitrogen). Taqman gene expression probes for *Dhh* (Mm01310203_m1), *Foxl2* (Mm00843544_s1), *Gng13* (Mm00458152_m1), *Lgr5* (Mm00438890_m1), *Ptgds* (Mm01330613_m1) and *Wnt4* (Mm00437341_m1) were purchased from Applied Biosystems. PCR was performed using the Step One Real-Time PCR System (Applied Biosystems). Relative levels of the transcripts were normalized to that of *Actb* (4351315; Applied Biosystems) or *Gapdh* (4351309; Applied Biosystems) as an endogenous reference.

### Tracing of surface epithelial/subepithelial cells using Qdot probes

To visualize the dynamics of the surface epithelium, gonadal surface epithelia were labelled using the Qtracker 655 Cell Labeling Kit (Q25029; Invitrogen). Briefly, whole gonads with mesonephros were treated with DT for 24 h and incubated with 10 nM Qdot probes in FCS-DMEM for 1 h. The labeled gonads were washed in culture medium several times and incubated in FCS-DMEM for 4 days. Some gonads were fixed immediately after labeling to ensure the initially labeled surface area. Cultured gonads were finally fixed and embedded in OCT compound. Cryosections were processed for immunohistochemistry as described above.

### Recombined culture of XY Tg gonad with GFP-positive mesonephros

Gonads were separated from the XY Tg at E11.5 (16-18 tail-somite stage). GFP-positive mesonephroi were dissected from the XY CAG-EGFP embryos at E11.5. After genotyping by PCR, each Tg gonad was assembled with GFP-positive mesonephros and cultured on 1.5% agar blocks in FCS-DMEM for 24 h to develop the combined testes correspond to E12.5 (Hiramatsu et al., 2009). These explants of the combined gonad-mesonephros were treated with DT for 24 h and recovery-cultured in FCS-DMEM for 4 days. Paraffin wax-embedded sections of these combined explants were co-stained using anti-FOXL2 and anti-GFP antibodies, as described above.

### Statistical analysis

Quantitative data are presented as mean±s.e.m. or as box-and-whisker plots. Two-tailed Student's *t*-test was performed for single comparisons between two groups. For more than two groups, one-way ANOVA followed by Dunnett's test or Tukey's test were performed. Statistical analysis was carried out using R (v. 3.4.3; R Development Core Team) software. *n* refers to the number of samples. *P*<0.05 was considered indicative of significance, and levels of significance are shown as *P<0.05 and **P<0.01.

## Supplementary Material

10.1242/develop.201660_sup1 Supplementary information
