# Supplementary material for: Partial male-to-female reprogramming of mouse fetal testis by Sertoli cell ablation
Source: Development. 2023 Jul 17;150(14):dev201660. doi: 10.1242/dev.201660 (PMC10399983; doi:10.1242/dev.201660)
Supplement: Supplementary information [file develop-150-201660-s1.pdf]

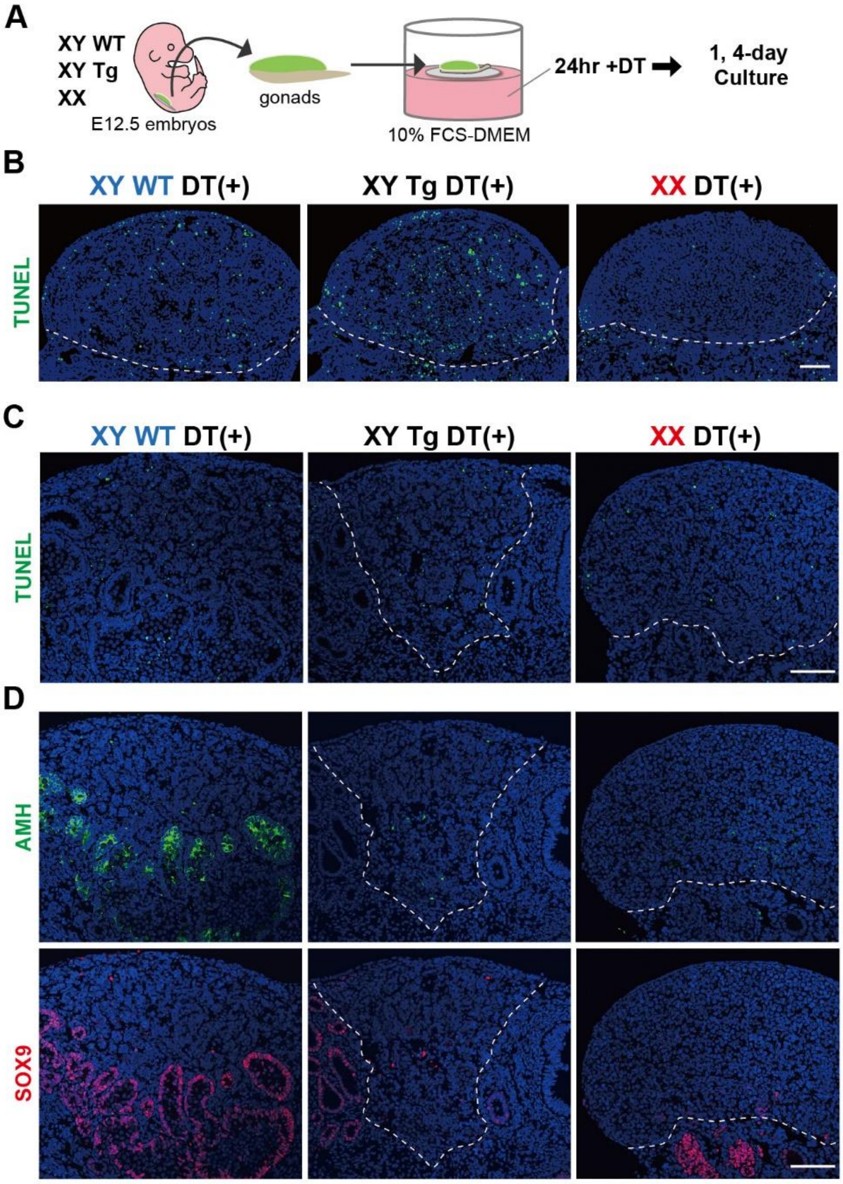

Fig.S1

Fig. S1. TUNEL, anti-AMH, and -SOX9 staining patterns in Tg testes after 4-day culture following diphtheria toxin (DT) treatment.

(**A**) Testes isolated from Tg embryos and WT littermates at E12.5 were cultured in FCS-DMEM for 1 day or 4 days following 24 h DT treatment. (**B, C**) TUNEL (green) staining of DT-treated explants in 1-day (B) and 4-day (C) cultures, showed increased TUNEL signals in the degenerated testis cords of the Tg explants in 1-day culture, but not in 4-day culture. **(D)** Anti-AMH (green) and -SOX9 (red) immunofluorescence (DAPI, blue) of the DT-treated explants in 4-day culture, showing a complete depletion and no recovery of the fetal Sertoli cells in DT-treated Tg explants. Broken lines indicate the border between gonad and mesonephros. Scale bar, 100 µm.

Development • Supplementary information

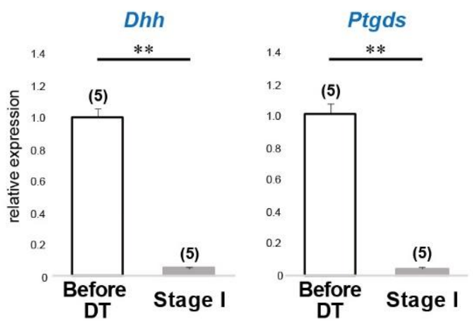

Fig.S2

Fig. S2. Altered transcript levels of *Dhh* and *Ptgds* before and after DT treatment in Tg testes at E12.5.

RT-qPCR analysis showed significantly reduced *Dhh* and *Ptgds* levels in 1-day culture of DT-treated Tg testes, as compared to the control testes before DT treatment (**$P < 0.01$ by unpaired Student's *t*-test). *Actb* was used as the endogenous reference. Data are presented as means ± SEM. Numbers in parentheses indicate the number of samples.

Development • Supplementary information

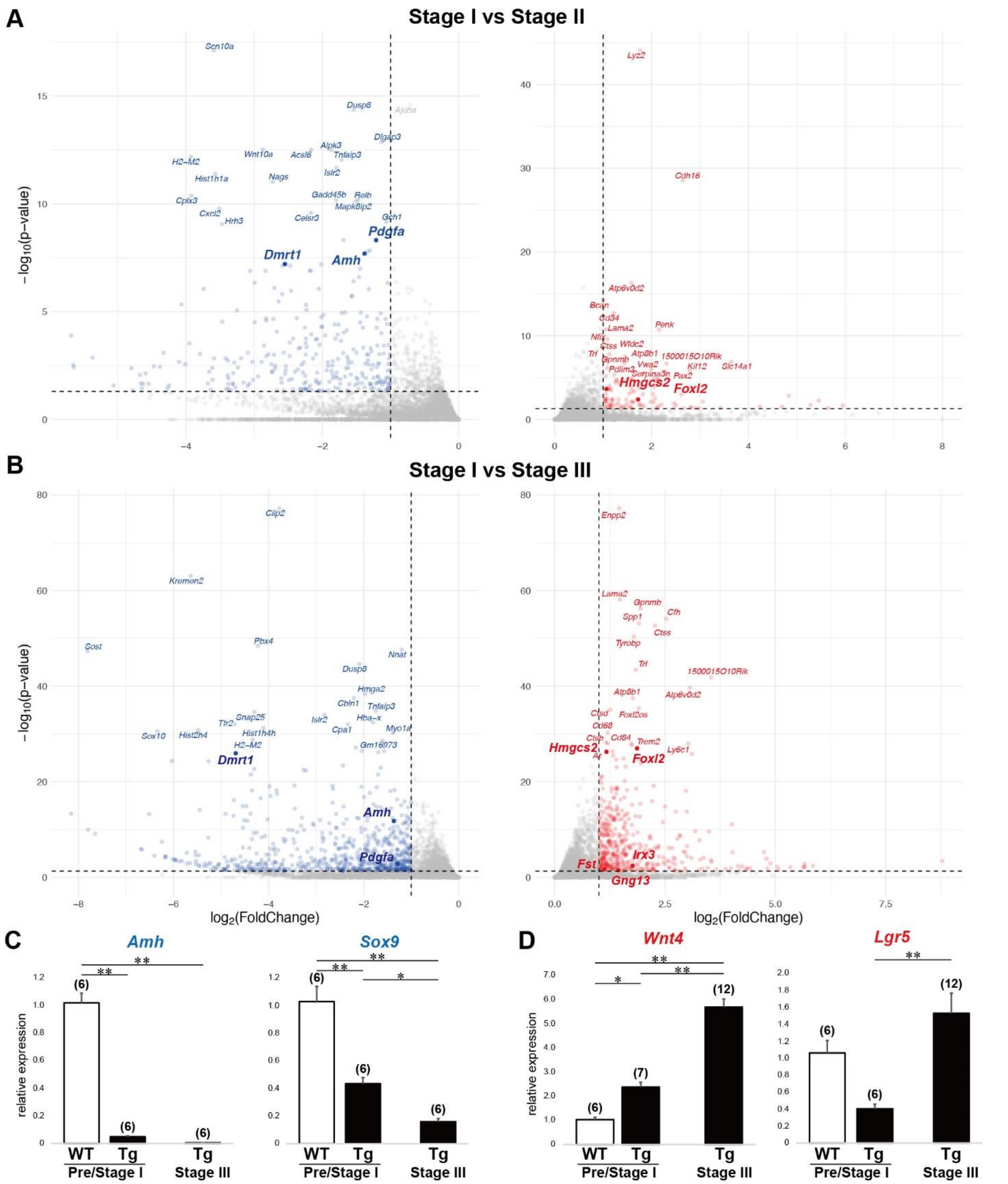

Fig.S3

Fig. S3. Differentially expressed genes (DEGs) and induced Wnt4 and Lgr5 expression in a 4-day culture of E12.5 testes with Sertoli cell ablation.

(A-B) Volcano plot of DEGs in stage II (A) and III (B) (horizontal broken lines, $-\log_{10}(0.05)$ and vertical broken lines, $|\log_2(\text{fold-change})| = 1$; relative to stage I). The top 20 genes ranked by modified *P*-value are indicated by symbols (bold symbols, testis- or ovary-specific marker genes with significant differences). **(C-D)** RT-qPCR analysis showed significantly increased *Wnt4* and *Lgr5* expression, together with reduced *Amh* and *Sox9* expression, in Tg testes in 4-day culture after 24 h DT treatment corresponding to stage III, compared to DT-treated WT and Tg testes before culture (**P* < 0.05, ***P* < 0.01; one-way ANOVA followed by the Tukey test). Data are means ± SEM. *Gapdh* was used as the endogenous reference. Numbers in parentheses are numbers of samples.

Development • Supplementary information

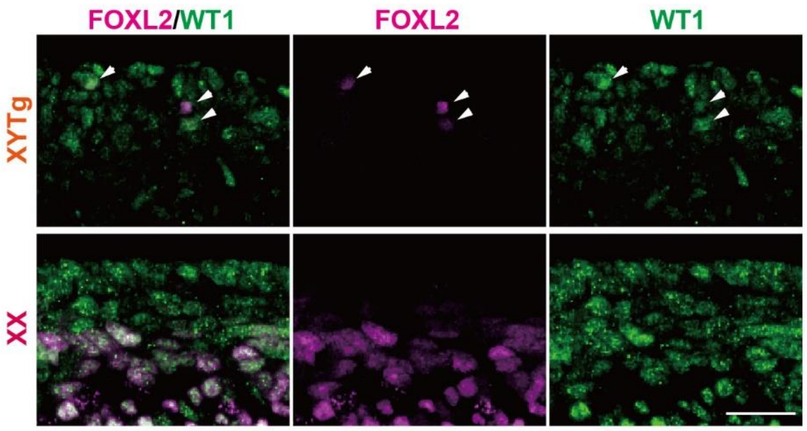

Fig.S4

Fig. S4. Anti-FOXL2 and -WT1 staining patterns in the Tg testis and ovary after 4-day culture following DT treatment.

Anti-FOXL2 (magenta) and -WT1 (green) immunofluorescence of DT-treated Tg explants, showing FOXL2-positive signals (arrowheads) in the WT1-positive subepithelial cell clusters. Scale bar, 25 µm.

Development • Supplementary information

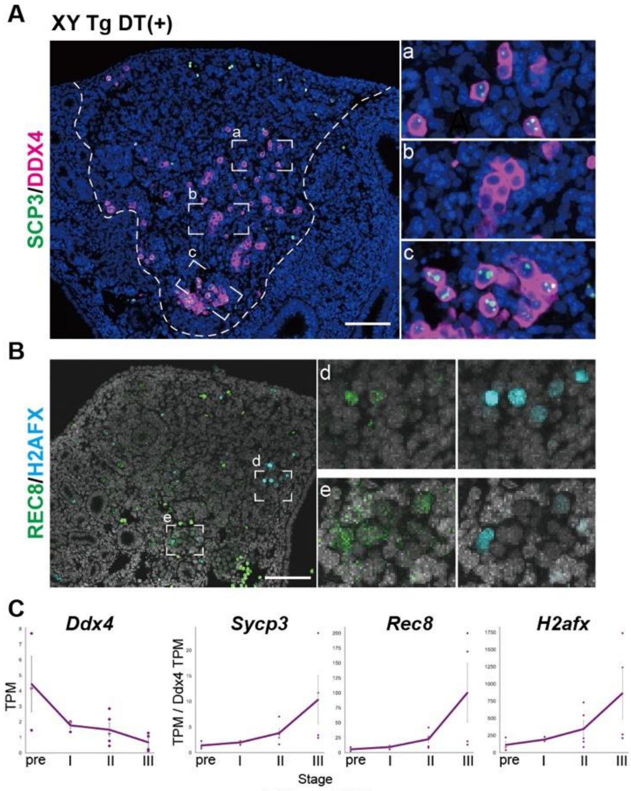

Fig.S5

Fig. S5. Meiotic initiation in germ cells survived in DT-treated Tg testis.

(A, B) Anti-DDX4 (a germ cell marker; magenta) and -SCP3 (a meiotic marker; green) (A) and anti-REC8 and -H2AFX (meiotic markers; green and cyan) (B) immunofluorescence of Tg testes in 4-day culture after DT treatment (DAPI, white). A few DDX4-positive germ cells are found to survive in DT-treated testes, with some of their nuclei being positive for SCP3, REC8, and H2AFX. Broken lines indicate the border between the gonad and mesonephros. The right panels (a-e) show high magnification images of the regions surrounded by broken rectangles. Scale bar, 100 µm. **(C)** Relative expression levels of three meiotic marker genes, namely *Sycp3*, *Rec8* and *H2afx*, relative to *Ddx4* (TPM per *Ddx4* TPM from bulk RNA-seq data, mean ± SEM on the y-axis) at each stage (x-axis).

Development • Supplementary information

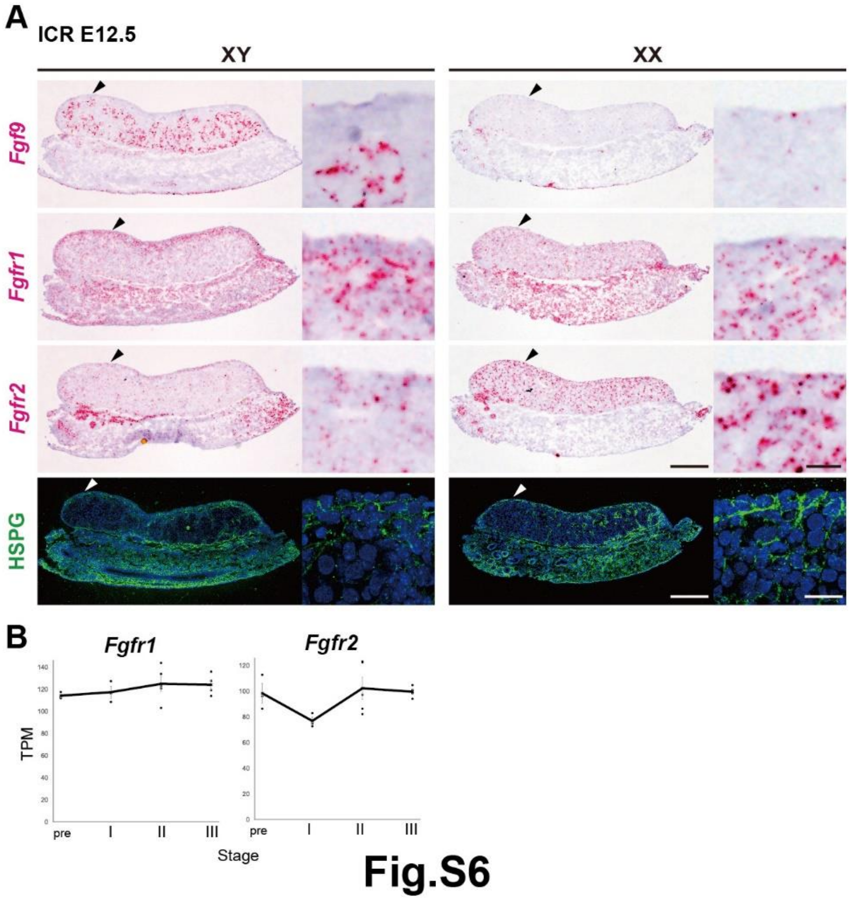

Fig. S6. *Fgfr1*, *Fgfr2*, and heparan sulfate proteoglycan (HSPG) are expressed in the surface epithelia/subepithelia and peri-mesonephric stroma of E12.5 testes *in vivo*.

(A) *In situ* hybridization of *Fgf9*, *Fgfr1*, and *Fgfr2* and anti-HSPG immunofluorescence images of E12.5 WT testes (left) and ovaries (right) *in vivo*. In contrast to male-specific *Fgf9* signals in testis cords, *Fgfr1* and *Fgfr2*, as well as HSPG, are expressed mainly in the surface epithelial/subepithelial and the peri-mesonephric regions, outside the testis cords, in testes at E12.5. Right panels show high magnification images of the surface epithelial/subepithelial region (arrowheads). Scale bars, 200 µm and 20 µm in the left and right panels, respectively. **(B)** Relative expression levels of *Fgfr1* and *Fgfr2* (TPM from bulk RNA-seq data, mean ± SEM on the y-axis) at each stage (x-axis).

Development • Supplementary information

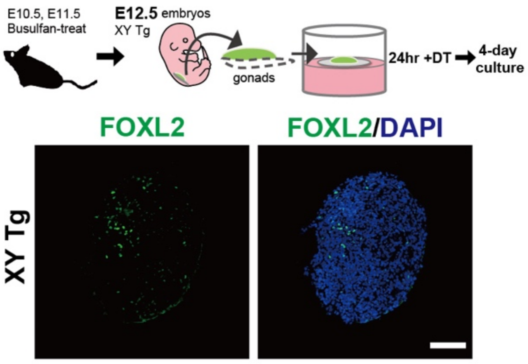

Fig. S7

Fig. S7. Anti-FOXL2 immunofluorescence in the busulfan-pretreated Tg testis after 4-day culture following DT treatment.

Testes without the adjacent mesonephros were isolated from E12.5 Tg embryos pretreated with busulfan, and cultured for 4 days following 24 h DT treatment. Anti-FOXL2 immunofluorescence of these explants showed the ectopic appearance of FOXL2-positive cells in a similar manner to those in busulfan non-treated Tg testes. Scale bar, 100 µm.

Development • Supplementary information

Table S1. List of stage-dependently transcript-changed genes in DT-treated Tg testes classified by k-Mean clustering (related to Fig 2).

(A) 750 stage-dependently transcript-reduced genes in DT-treated Tg testes classified by k-Mean clustering. (B) 546 stage-dependently transcript-increased genes in DT-treated Tg testes classified by k-Mean clustering

Click here to download Table S1

Table S2. List of stage-dependently differentially expressed genes (DEGs) in DT-treated Tg testes, which are shared with sex-dimorphic genes (related to Fig. S3).

(A) 154 genes shared between DEGs (Stage I > III) and E12.5, E13.5 testis-specific genes. (B) 39 genes shared between DEGs (Stage I < II) and E12.5, E13.5 ovary-specific genes. (C) 102 genes shared between DEGs (Stage I < III) and E12.5, E13.5 ovary-specific genes

Click here to download Table S2

Table S3. List of antibodies used in this study.

Antigen	Dilution	Description	Company	CAT#
AMH	1/200	Goat polyclonal	Santa Cruz	sc6886
DDX4 (MVH)	1/1000	Rabbit polyclonal	Abcam	ab13840
FOXL2	1/200	Goat polyclonal	Abcam	ab5096
GFP	1/200	Rabbit polyclonal	MBL	598
HSPG	1/100	Mouse monoclonal	Amsbio	10E4; 370255-1
H2AFX	1/500	Mouse monoclonal	Millipore	05-636
NR2F2	1/200	Mouse monoclonal	Perseus Proteomics	PP-H7147-00
PAX8	1/10	Mouse monoclonal	Abcam	ab53490
REC8	1/200	Rabbit monoclonal	Abcam	ab192241
SCP3	1/500	Mouse monoclonal	Santa Cruz	sc74569
SOX9	1/10000	Rabbit polyclonal	Merk Millipore	ab5535
WT1	1/300	Rabbit monoclonal	Abcam	ab89901
3βHSD	1/1000	Goat polyclonal	Santa Cruz	sc30821
3βHSD	1/1000	Rabbit polyclonal	TransGenic Inc.	KO607
Goat IgG (H+L)	1/200	Horse polyclonal biotinylated	Vector Laboratories	BA-9500
Rabbit IgG (H+L)	1/200	Goat polyclonal biotinylated	Vector Laboratories	BA-1000
Mouse IgG (H+L)	1/200	Horse polyclonal biotinylated	Vector Laboratories	BA-2001
Goat IgG (H+L)	1/200	Chicken polyclonal Alexa 488 conjugated	Invitrogen	A-21467
Goat IgG (H+L)	1/200	Donkey polyclonal Alexa 594 conjugated	Invitrogen	A-11058
Rabbit IgG (H+L)	1/200	Chicken polyclonal Alexa 488 conjugated	Invitrogen	A-21441
Rabbit IgG (H+L)	1/200	Chicken polyclonal Alexa 594 conjugated	Invitrogen	A-21442
Mouse IgG (H+L)	1/200	Chicken polyclonal Alexa 488 conjugated	Invitrogen	A-21200
Mouse IgG (H+L)	1/200	Chicken polyclonal Alexa 594 conjugated	Invitrogen	A-21201
Mouse IgG (H+L)	1/200	Donkey polyclonal Alexa 647 conjugated	Abcam	ab150107

Development • Supplementary information
